# Isoform-Specific Lysine Methylation of RORα2 by SETD7 Is Required for Association of the TIP60 Coactivator Complex in Prostate Cancer Progression

**DOI:** 10.3390/ijms21051622

**Published:** 2020-02-27

**Authors:** Hyerin Song, Jung Woong Chu, Su Chan Park, Hyuntae Im, Il-Geun Park, Hyunkyung Kim, Ji Min Lee

**Affiliations:** 1 Department of Molecular Bioscience, College of Biomedical Sciences, Kangwon National University, Chuncheon 24341, Korea; shyerin521@gmail.com (H.S.); psc4932@naver.com (S.C.P.); limht95@naver.com (H.I.); dlfrms2382@naver.com (I.-G.P.); 2 Department of Biochemistry and Molecular Biology, Korea University College of Medicine, Seoul 02841, Korea; cnwjddnd932@korea.ac.kr

**Keywords:** RORα2, oncogene, prostate cancer, N-terminal domain, lysine methylation

## Abstract

The retinoid acid-related orphan receptor α (RORα), a member of the orphan nuclear receptor superfamily, functions as an unknown ligand-dependent transcription factor. RORα was shown to regulate a broad array of physiological processes such as Purkinje cell development in the cerebellum, circadian rhythm, lipid and bone metabolism, inhibition of inflammation, and anti-apoptosis. The human *RORα* gene encodes at least four distinct isoforms (RORα1, -2, -3, -4), which differ only in their N-terminal domain (NTD). Two isoforms, RORα2 and 3, are not expressed in mice, whereas RORα1 and 4 are expressed both in mice and humans. In the present study, we identified the specific NTD of RORα2 that enhances prostate tumor progression and proliferation via lysine methylation-mediated recruitment of coactivator complex pontin/Tip60. Upregulation of the RORα2 isoform in prostate cancers putatively promotes tumor formation and progression. Furthermore, binding between coactivator complex and RORα2 is increased by lysine methylation of RORα2 because methylation permits subsequent interaction with binding partners. This methylation-dependent activation is performed by SET domain containing 7 (SETD7) methyltransferase, inducing the oncogenic potential of RORα2. Thus, post-translational lysine methylation of RORα2 modulates oncogenic function of RORα2 in prostate cancer. Exploration of the post-translational modifications of RORα2 provides new avenues for the development of tumor-suppressive therapeutic agents through modulating the human isoform-specific tumorigenic role of RORα2.

## 1. Introduction

Retinoid acid-related orphan receptor α (RORα) belongs to the nuclear receptor family 1 group F members (NR1F) and is classified as an orphan nuclear receptor because endogenous ligands are not yet determined [1,2,3]. The messenger RNA (mRNA) and protein isoforms generated by alternative processing of primary RNA transcripts may differ in protein function, structure, localization, or other biological properties. By this alternative exon splicing of mRNA, the *RORα* gene generates four isoforms that have a common DNA-binding domain (DBD) and ligand-binding domain (LBD), but contain distinct N-terminal domains (NTDs) in humans [4,5]. All isoforms share similar amino-acid sequences but are characterized by distinct NTDs generated by alternative RNA processing. NTD and zinc finger motifs in the DBD function in concert to provide specific DNA-binding properties to the RORα isoforms. RORα1 and RORα4 are present ubiquitously, whereas the expression pattern of RORα2 and RORα3, isoforms that exist only in humans, is tissue- and cell-type-specific. RORα binds as a monomer or homodimer to a specific DNA sequence known as the ROR response element (RORE) that consists of a 6-bp A/T-rich sequence preceding a half-site core motif PuGGTCA [6,7]. RORα was reported to regulate transcription of target genes through its interactions with many coactivators and corepressors, and it was shown to play important roles in many pathophysiological processes including circadian rhythm, development, the immune system, and metabolic homeostasis [5,8,9,10,11]. Moreover, recent studies demonstrated that RORα is involved in tumorigenesis, suggesting that RORα may be considered a potential therapeutic target in many cancers [12,13,14,15]. Post-translational modification and interaction with coregulators are pivotal mechanisms via which orphan nuclear receptor activity can be modulated in a ligand-independent manner [16,17]. In particular, several studies revealed that the distinct NTD, which differs between the RORα isoforms, provides sites for coregulator binding and protein modification so that each isoform functions as a potent regulator to activate target gene expression under different physiological conditions. 

Prostate cancer (PCa) is common cancer with a high incidence of mortality in men [18,19]. Family history, levels of steroid hormone, age, and ethnicity are known risk factors, and inhibition of androgen signaling is the gold-standard treatment. While detection is now more precise, and treatment is available, PCa incidence in many countries increased, underscoring a need for the detailed molecular mechanisms of PCa to be further elucidated [20,21]. Recently, growing evidence suggested that the nuclear receptor superfamily plays a role in the tumorigenesis of PCa and treatment resistance [22]. Vitamin D receptor and farnesoid X receptors function as tumor suppressors [23,24], while androgen receptor, as well as glucocorticoid receptor, augment tumorigenesis [25]. Intriguingly, RORα1 also attenuated cell proliferation and invasive potential in PCa [26]. However, since RORα family members show various regulatory mechanisms, and since these differences may be due to their distinct structure of all isoforms, a better understanding of the precise regulatory mechanism among RORα isoforms in PCa progression will help to develop new prevention approaches. 

In this study, we investigated how human-specific RORα2 functions as an essential factor to promote cell proliferation and clonogenic growth rates in the PCa cells. We identified pontin/Tip60 as a coactivator complex and reptin as a corepressor that regulates expression of RORα2 target genes, as well as revealing that RORα2 is methylated by SET domain containing 7 (SETD7) and demethylated by jumonji C (JmjC)-domain-containing histone demethylase 3A (JHDM3A). Unlike RORα1, which is methylated and degraded by enhancer of zeste homolog 2 (EZH2), methylation of RORα2 contributes to increased target gene expression and tumorigenesis by enhancing binding affinity with coactivators [13]. Taken together, our data highlight the mechanism via which methylated RORα2 promotes the oncogenic properties of human PCa cells. This finding will lead to the development of new therapeutic strategies in PCa.

## 2. Results

### 2.1. RORα2 Functions as a Selective Oncogene in PCa

**Figure 1:**
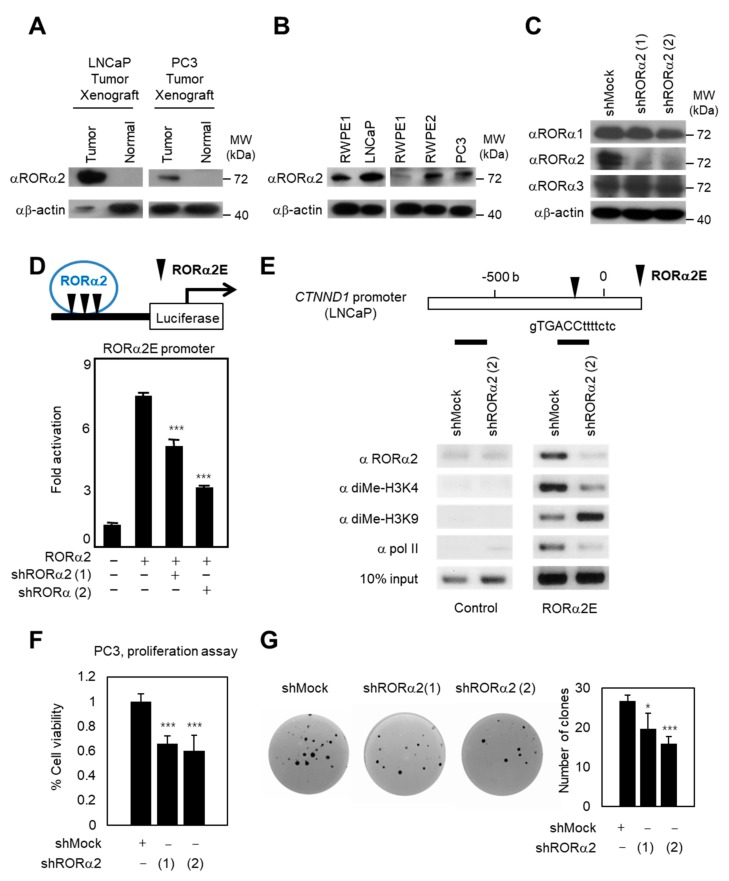
Retinoid acid-related orphan receptor α (RORα2) increases the proliferation and growth of prostate cancer (PCa) cells. (**A**) Lysates of xenograft tumors from LNCaP or PC3 cells were subjected to immunoblot analysis for RORα2 expression. (**B**) Expression of RORα2 in normal (RWPE1) and prostate cancer cell lines as assessed by immunoblotting. (**C**) Efficacy and specificity of knockdown by two individual short hairpin RNAs (shRNAs) against RORα2 are shown by immunoblot analysis against RORα1, 2, and 3 antibodies. (**D**) Introduction of shRORα2 decreased transcriptional activation of the RORα2E-luciferase reporter. Data are represented as means ± SD for three independent experiments. Statistical significance was calculated by a two-tailed, unpaired *t*-test (*** *p* < 0.001). (**E**) ChIP assay on the *CTNND1* promoter luciferase reporter in LNCaP cells with or without shRNA of RORα2. shRORα2 (2) was used as representative for this assay, and occupancy of the control or RORα2E in *CTNND1* promoter by RORα2, di-methyl H3K4, di-methyl H3K9, and polymerase II was analyzed. (**F**) The MTS cell proliferation assay of PC3 cells expressing shMock or shRORα2. MTS absorbance was determined at 490 nm. (**G**) Photographs from the clonogenic assay of PC3 cells expressing shRNA against RORα2. The number of colonies was quantified in control and shRORα2-expressing PC3 cells, as shown in the right panel. Statistical significance was calculated by a two-tailed, unpaired *t*-test (* *p* < 0.05, *** *p* < 0.001).

To define the unknown roles of RORα2 isoform in PCa, we examined the expression of RORα2 in tumorigenesis experiments with prostate cells in athymic nude mice. LNCaP and PC3 are representative PCa cell lines; LNCaP is lymph node metastasis-derived, and PC3 is bone metastasis-derived. To validate whether the expression levels of RORα2 are correlated with cancer progression and metastatic potential or not, RORα2 protein levels in xenograft tumors of LNCaP and PC3 were compared with their normal counterparts. Western blots revealed low or no expression of RORα2 in normal tissues, and a significant upregulation in tumor tissues (Figure 1A). In support of oncogenic roles of RORα2 in PCa, immunoblotting analysis showed that RORα2 expression was dramatically increased in prostate metastatic cancer cells such as LNCaP and PC3 compared to that in a normal prostate cell line such as RWPE1 (Figure 1B). RORα2-dependent target genes were tested using short hairpin RNA (shRNA) against RORα2 to confirm the oncogenic potential of the RORα2 isoform. The shRNA against RORα2 reduced its levels, but did not reduce levels of RORα1 or RORα3, as indicated by immunoblot analysis (Figure 1C). Indeed, knockdown of RORα2 reduced downstream target genes (Figure 1D) and decreased recruitment of RORα2 on *CTNND1* promoter (Figure 1E). Previously, we identified *CTNND1* as an RORα2-specific target gene in breast cancer cells [12]. To examine whether RORα2 is recruited on *CTNND1* promoter and further increases oncogenic potential in PCa cells, a chromatin immunoprecipitation (ChIP) assay was performed in the presence or absence of shRNA against RORα2. RORα2 was recruited to *CTNND1* promoter along with di-methyl H3K4 and RNA polymerase II (Figure 1E). However, knockdown of RORα2 nearly abolished the recruitment of di-methyl H3K4 and polymerase II, indicating that activation of histone marker recruitment to the *CTNND1* promoter is mediated by RORα2 in PCa cells. In contrast, the recruitment of repressive histone marker di-methyl H3K9 was increased by knockdown of RORα2 on the *CTNND1* promoter. Our data indicate that the oncogenic potential of RORα2 is critical in PCa cells. 

As upregulation of RORα2 is correlated with cell growth, proliferation, and invasiveness in PCa, we next explored whether the expression of RORα2 could increase cellular proliferation in PCa cells. A proliferation assay, which measures the increase in cell viability over 48 h for shRORα2-expressing PC3 cells as well as control PC3 cells, revealed an inverse correlation between the proliferation rate and the RORα2 expression levels (Figure 1F). To confirm whether RORα2 could further stimulate anchorage-independent growth, PC3 cells stably expressing control shRNA and two independent shRNAs of RORα2 were examined for colony formation, an important property of tumor cell growth (Figure 1G). Introduction of shRORα2 decreased the number of colonies and these results collectively supported our hypothesis that endogenous RORα2 protein plays a critical role in tumorigenesis of PCa.

### 2.2. RORα2 Selectively Binds to Pontin/Tip60 Coactivator Complex and Reptin Corepressor

**Figure 2:**
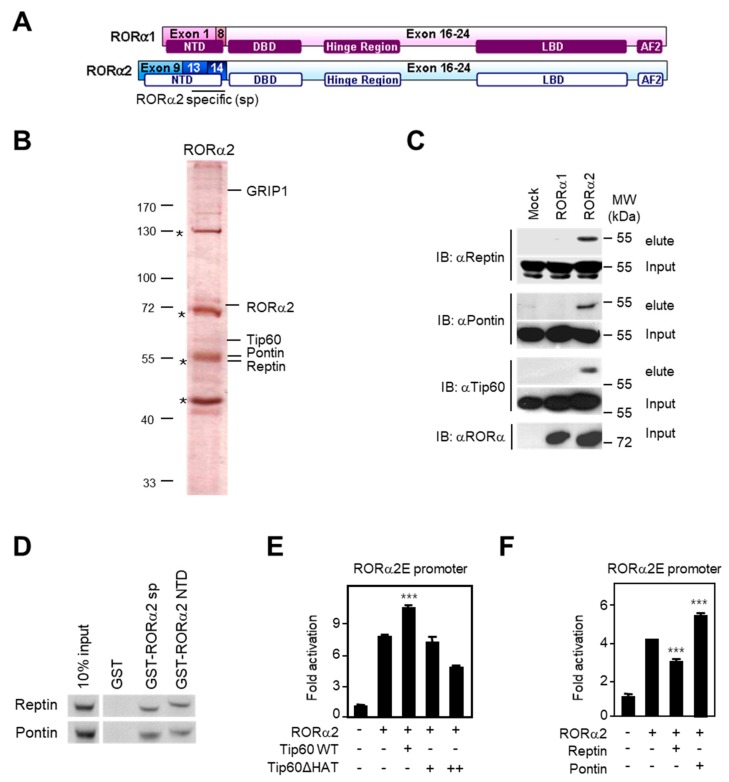
RORα2 complexes incorporate pontin/Tip60 and reptin through RORα2-specific N-terminal domain (NTD). (**A**) Schematic presentation of the RORα1 and RORα2 isoforms. NTDs of RORα1 and RORα2 are significantly different. (**B**) RORα2-binding proteins were purified from extracts obtained from HEK293 cells stably expressing Flag-tagged RORα2. The bound proteins were resolved by SDS-PAGE and prepared for LC-MS/MS analysis. (**C**) Western blot analysis indicates that RORα2 specifically binds to reptin, pontin, and Tip60. (**D**) Glutathione S-transferase (GST) pulldown assay shows the direct interaction of GST–RORα2 sp (Exon 13 and 14) or GST-RORα2 NTD (Exon 9, 13, and 14) with reptin and pontin. (**E** and **F**) Luciferase reporter assays were conducted after co-transfection with RORα2E luciferase reporter. Expression of Tip60 activated RORα2E-luciferase reporter, but reporter was not activated by Tip60ΔHAT, an acetyltransferase enzymatic deletion mutant (**E**). Co-transfection of RORα2 and Tip60 WT significantly increased reporter activities than RORα2 only transfected control (*** *p* < 0.001). Expression of reptin repressed RORα2E luciferase reporter, whereas elevated pontin induced reporter (**F**). Data are represented as means ± SD for three independent experiments.

We originally reported that RORα1 functions as a tumor suppressor in prostate, breast, and colon cancers [13,14,15,26]. Since RORα2 has an NTD distinct from RORα1, we conducted complex purification of RORα2 (Figure 2A). To investigate the functional modules of RORα2, we employed a FLAG epitope-tag (peptide sequence DYKDDDK) strategy and attempted to purify binding proteins for RORα2. Liquid chromatography–mass spectrometry/mass spectrometry (LC–MS/MS) identified binding proteins for RORα2 purified from the FLAG M2 affinity column (Figure 2B and Figure S1, Supplementary Materials). While both RORα1 and RORα2 interact similarly with several coregulators, the profiles of RORα2 NTD-mediated complexes were substantially different (Figure 2B). Binding to Grip1, common interactor partners for RORα1 and RORα2, was detected in both RORα1- and 2-purified elutes. In contrast, RORα2-bound samples demonstrated selective linkage between the reptin/pontin/Tip60 complex, as shown in Figure 2C. These RORα2-specific binding partners were co-purified with FLAG–RORα2, not with FLAG–RORα1 in 293T cells. Consistent with this observation, direct binding of reptin and pontin with the NTD domain of RORα2 was confirmed by glutathione S-transferase (GST) pulldown assay (Figure 2D). RORα2 and RORα3 share the first exon (Exon 9), but exon 13 and 14 only exist in the RORα2 human isoform. We also confirmed the exclusive binding between RORα2 and pontin/reptin using RORα2 sp (specific for RORα2, not for RORα3, including Exon 13 and 14) constructs (Figure 2D). Furthermore, introduction of RORα2 and Tip60, which has a histone acetyltransferase (HAT) domain, led to activation of the downstream signaling pathway (Figure 2E). In support of this idea, reptin potentiated RORα2-dependent transcriptional repression, whereas pontin stimulated activator function of RORα2 on target gene promoters (Figure 2F). Together, the coactivator function of pontin/Tip60 and the corepressor activity of reptin on RORα2 target gene promoters require binding of the complex to RORα2 via its distinct NTD. 

### 2.3. Lysine Methylation of RORα2 by SETD7 Is Crucial for Downstream Target Gene Activation

**Figure 3:**
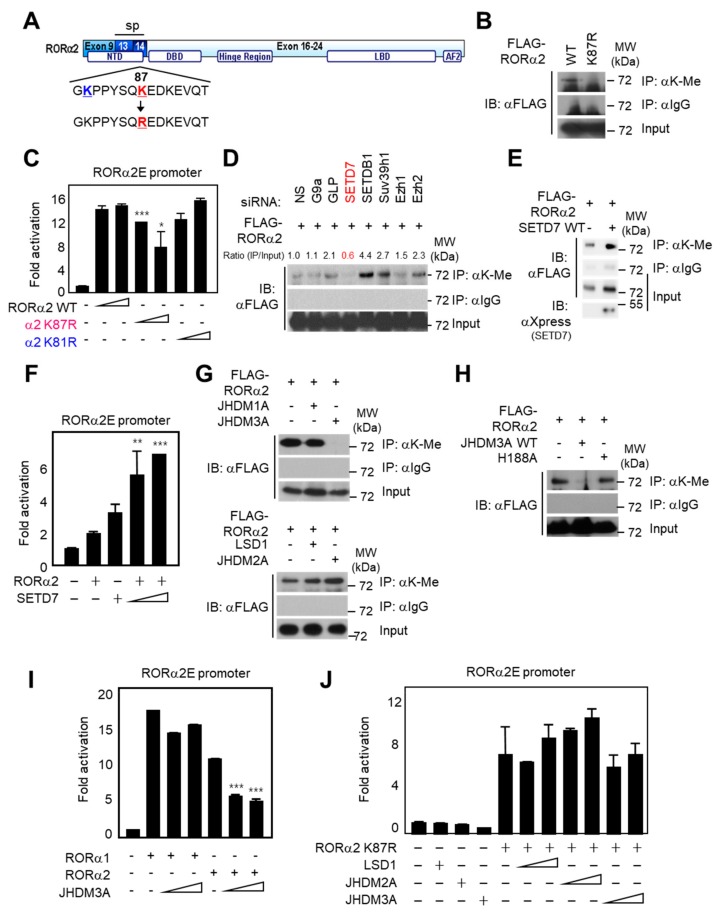
Lysine 87 of RORα2 is crucial for methylation by SETD7. (**A**) Schematic representation of the NTD of RORα2. The position of the K87R mutation is highlighted in red, and control mutation lysine 81 site is highlighted in blue. (**B**) 293T cells were transfected with FLAG–RORα2 WT or K87R mutant and cell lysates were immunoprecipitated with anti-lysine (K) methyl antibody, followed by immunoblotting analysis against anti-FLAG antibody indicating methylated RORα2. (**C**) Introduction of RORα2 K87R mutant failed to increase the transcriptional activation of the RORα2E luciferase reporter. Data are represented as means ± SD for three independent experiments. Statistical significance was calculated by a two-tailed, unpaired *t*-test (* *p* < 0.05, ** *p* < 0.01, *** *p* < 0.001). (**D**) RORα2 methylation was detected after co-transfection with small interfering RNAs (siRNAs) of each methyltransferase and revealed that knockdown of SETD7 reduced methylated levels of RORα2. (**E**) Hismax-SETD7 was transfected with FLAG–RORα2, and, after immunoprecipitation with anti-lysine antibody, the methylation level of RORα2 was detected by anti-FLAG antibody. (**F**) Introduction of SETD7 increased the transcriptional activation with RORα2 in a dose-dependent manner. (**G**) 293T cells were transfected with JHDM1A, 2A, 3A, or LSD1, and cell extracts were subject to immunoprecipitation with anti-lysine methyl antibody, followed by immunoblotting against FLAG to detect FLAG–RORα2 methylation status. JHDM3A abolished lysine methylation of RORα2. (**H**) 293T cells were transfected with either JHDM3A WT or H188A enzymatic-dead mutant, and the cell extracts were subjected to immunoblot analysis to detect RORα2 methylation. (**I**) Introduction of JHDM3A decreased the transcriptional activation of the RORα2E luciferase reporter with RORα2 selectively. Compared to RORα2, RORα1 transcriptional activities were not affected by JHDM3A expression. (**J**) Transcriptional activities of RORα2 K87R methylation mutant were not affected by expression of LSD1, JHDM2A, and JHDM3A. Data are represented as means ± SD for three independent experiments.

While some methyltransferases were shown to act on transcription factors, as well as histones [27,28], the possibility of selective substrate recognition specificity among isoforms was not extensively studied. We used MeMo software by Health Sciences Library System, a web tool for prediction of protein methylation modifications. This analysis predicted the K87 site of RORα2 as a “hit target” for methylation (Figure 3A). This distinct site is not conserved in RORα1, which has a different NTD than RORα2. Therefore, we hypothesized that the selective sequence in the NTD of RORα2 allows its methylation by a unique methyltransferase, different from the EZH2 methyltransferase of RORα1 [13]. Firstly, we generated a K87R mutant in which a lysine residue was replaced by an arginine to abrogate lysine methylation. Co-immunoprecipitation assay with anti-lysine methyl antibody revealed that K87R mutation abolished RORα2 methylation, suggesting that K87 is the major lysine methylation site of RORα2 (Figure 3B). To further examine whether the RORα2-mediated regulation of downstream target genes is affected by RORα2 lysine methylation, we performed a luciferase assay with the introduction of RORα2 wild type (WT), K81R (control mutant), or K87R. Surprisingly, only RORα2 K87R exhibited diminished target gene activation, whereas WT and K81R mutant resulted in increased transcriptional activities (Figure 3C). These data demonstrate that lysine-methylation dependent activation of RORα2 is responsible for methylation at the K87 site.

To assess which methyltransferase and demethylase determine the methylation status of RORα2, knockdown of methyltransferases by specific siRNA was tested by methylation assay for RORα2. Immunoprecipitation assay confirmed that knockdown of SETD7 predominantly failed to be recognized by lysine-methylated RORα2 (Figure 3D). We confirmed SETD7-mediated lysine methylation of RORα2 by overexpressing SETD7 in vitro (Figure 3E). Consistent with the finding that SETD7 potentiates methylation of RORα2 specifically, overexpression of SETD7 was sufficient to further activate the RORα2E-containing promoter activity, whereas other methyltransferases failed to further activate RORα2E luciferase activity (Figure 3F and Figure S2, Supplementary Materials). Introduction of demethylase JHDM3A WT reduced methylation levels of RORα2 whereas JHDM1A, JHDM2A, lysine-specific histone demethylase 1A (LSD1), and JHDM3A enzymatic mutant (H188A) failed to decrease methylation status (Figure 3G,H). As expected, JHDM3A is the specific demethylase of RORα2, and not of RORα1, thus confirming that the RORα2-specific demethylation by JHDM3A diminished its transcriptional activities (Figure 3I). However, overexpression of JHDM3A failed to decrease RORα2E luciferase reporter activity with RORα2 K87R methylation mutant (Figure 3J). Taken together, these data suggest that SETD7-dependent methylation of RORα2 triggers increased downstream target gene activation.

### 2.4. Methylation of RORα2 by SETD7 Alters the Binding Affinity of a Coactivator Complex and Increases Tumorigenesis in PCa

**Figure 4:**
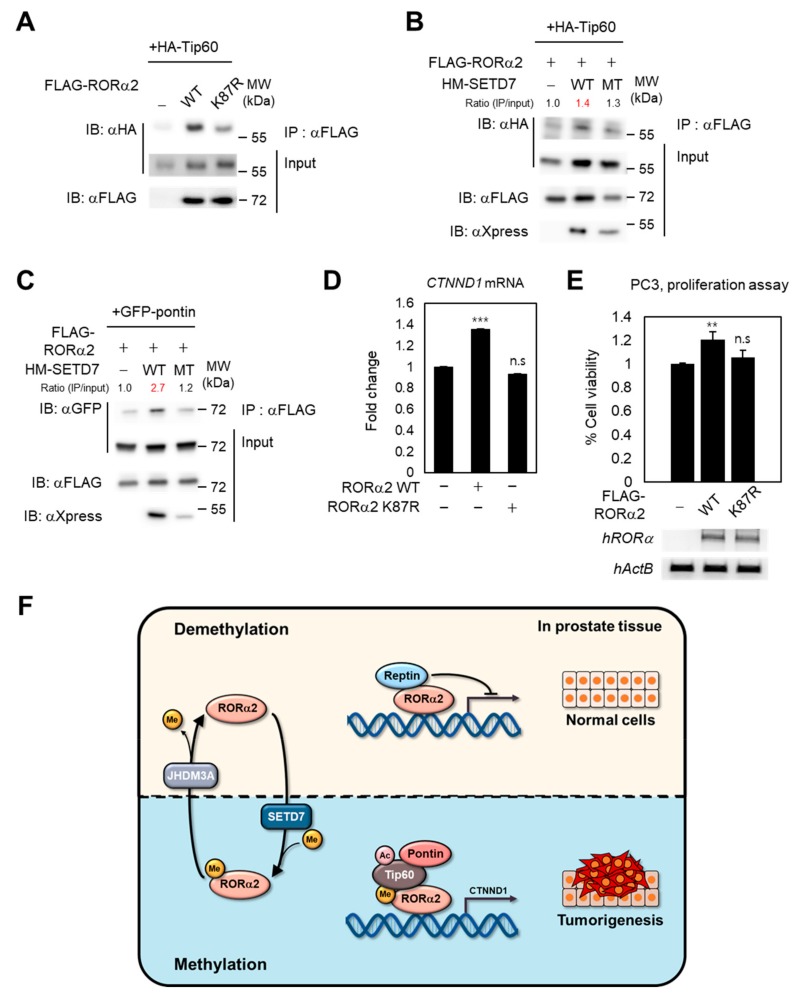
Tip60 coactivator interacts with RORα2 in a methylation-dependent manner and activates the signaling pathway downstream of RORα2. (**A**) Methylation of RORα2 induces its binding with Tip60. A binding affinity assay between Tip60 and RORα2 WT or K87R mutant was performed. (**B**,**C**) Cells were transfected with Hismax-SETD7 WT or enzymatic mutant and HA-Tip60 (B) or GFP-pontin (C), and the cell extracts were immunoprecipitated with anti-FLAG antibody followed by immunoblotting against anti-HA (B) or anti-GFP (C) antibodies in the presence or absence of enzymatic activities of SETD7. (**D**) Quantitative RT-PCR analysis of *CTNND1* transcripts was performed in 293T cells (mean ± SD, *n* = 3). Statistical significance was calculated by a two-tailed, unpaired *t*-test (** *p* < 0.01, *** *p* < 0.001, n.s = not significant). (**E**) The MTS cell proliferation assay of PC3 cells expressing RORα2 WT or RORα2 K87R. MTS absorbance was determined at 490 nm. Overexpressed levels of FLAG–RORα2 WT or RORα2 K87R were confirmed by RT-PCR (bottom). (**F**) Schematic model of activation of RORα2 target genes by SETD7-dependent methylation of RORα2.

Since lysine methylation of RORα2 induced the activation of the target gene, we examined whether methylation of RORα2 could trigger its transcriptional activities through increased interaction with coactivator complex pontin/Tip60. The lysine methylation mutant of RORα2 exhibited weak binding to Tip60, whereas RORα2 WT exhibited strong binding to Tip60 (Figure 4A). Consistent with these data, binding of RORα2 to Tip60 was increased by induction of SETD7 methyltransferase (Figure 4B). Failure of RORα2 methylation by SETD7 enzymatic mutant abrogated the binding of RORα2 to Tip60, confirming that methylation of the K87 site of RORα2 is crucial for binding to pontin/Tip60 coactivator complex. These data clearly demonstrate that SETD7-dependent methylation of RORα2 modulates the binding affinity of RORα2 toward coactivators. To further examine whether RORα2-mediated activation of target genes is affected by RORα2 methylation that leads to increased binding to pontin/Tip60, mRNA expression levels of RORα2 target gene *CTNND1*, involved in the signaling of prostate cancer progression, were detected after the introduction of either RORα2 WT or the lysine methylation mutant, K87R (Figure 4C). As expected, RORα2 WT increased mRNA levels of *CTNND1*, whereas RORα2 K87R resulted in decreased levels of *CTNND1*. These results indicate that RORα2 confers a transcriptional activator function on target gene promoters, which are related to cancer progression by the enhanced binding to coactivator complex via SETD7-dependent methylation on lysine 87 of RORα2 (Figure 4D).

To determine whether methylation of RORα2 is sufficient to support PCa cell proliferation, we tested the effects of RORα2 methylation status on cellular proliferation and growth of PC3 cells. The proliferation assay measured the increase in cell number over the course of 48 h for RORα2 WT and K87R mutant-expressing PC3 cells along with mock cells. Introduction of RORα2 WT increases the proliferation and growth of PC3 cells, whereas RORα2 K87R mutant did not stimulate cell proliferation (Figure 4E). These results suggest that methylation on RORα2 can augment the transforming potential of RORα2, consistent with our in vitro RORα2-dependent *CTNND1* transcriptional activation data. Collectively, we could conclude that SETD7 confers activation on RORα2-mediated target genes by enhanced binding to pontin/Tip60 complex via methylation on the K87 residue of RORα2, and it further stimulates RORα2-dependent tumorigenesis in PCa. This is possibly conversely regulated by reptin after JHDM3A-mediated demethylation of RORα2 (Figure 4F).

## 3. Discussion

In this manuscript, we identified a specific oncogenic signaling downstream pathway of RORα2: lysine methylation modification of RORα2 in modulation of binding with coactivator complex and PCa cell growth and proliferation. Given that coactivator complex pontin/Tip60 was obtained from RORα2 isoform-specific complexes, we wished to explore the possible roles of specific regulation of RORα2 [29]. We demonstrated that RORα2 is a direct substrate for SETD7 and that lysine methylation of RORα2 underlies transcriptional activation of RORα2 in the regulation of tumorigenic target genes in PCa cells, including *CTNND1*. In the event of methylation, both methylation and subsequent binding to coactivator complex are required steps for the coordinated regulation of this process. Given that JHDM3A demethylates RORα2, whereas SETD7 methylates RORα2, it is tempting to speculate that the two enzymes might work separately in certain biological processes that require dynamic methylation of RORα2 processes [17,30]. 

Our data show that methylation of RORα2 is responsible for the strong transcriptional regulatory function of RORα2 on target genes in the nucleus through its increased binding to pontin/Tip60 complex. It is perhaps surprising that only the RORα2 isoform was found to be methylated by its specific NTD and that methylation has such an impact on the modulation of oncogenic RORα2. RORα2 isoform-specific post-translational modification in PCa cells might represent a differential cancer-avoiding strategy by the RORα1 tumor suppressor, providing another layer of regulation and underscoring the importance of alternative splicing. 

Given that the methylation of non-histone substrate is involved in a variety of cellular processes, a link between lysine methylation and cancer can be anticipated [31,32,33]. SETD7 was suggested as a good candidate for drug targeting because it is the enzyme that methylates AR in PCa [17]. This might reflect close involvement of SETD7 in tumorigenesis by regulating methylation of various cellular targets, including RORα2 in PCa [34,35,36]. It is, therefore, tempting to explore the possibility that malignant progression of PCa cells might prefer methylated RORα2, utilizing either hyperactivation of SETD7 or, conversely, inactivation of JHDM3A [37,38]. 

In the present study, we provided evidence that lysine methylation of RORα2 is important for maintaining and exerting transcriptional activation processes with coactivator complex, and methylated RORα2 further led to an increase in proliferation and growth of PCa cells [39,40,41]. In contrast, RORα1, another major RORα isoform, was identified as a tumor suppressor crucial for conferring tumor-suppressive function in PCa [26,42]. We speculate that the lysine methylation status of certain proteins is a crucial modulator of cancer progression, and determining the upstream signal for the methylation of these proteins may shed light on the role of lysine methylation in human cancer [43,44,45]. However, these results have a limitation that clinical relevance was not directly verified using the specimens of prostate cancer patients. Therefore, further studies need to compare whether the methylation level of RORα2 is elevated in prostate cancer patients compare to the normal, which is helpful to prove the clinical significance of the oncogenic function of RORα2 in PCa. Elucidation of the biological importance of specific human protein methylations and their roles in cancer progression will provide information for understanding human cancer and developing human-specific therapeutic reagents [46,47,48,49]. 

## 4. Materials and Methods

### 4.1. Reagents

The following antibodies were purchased from Santa Cruz Biotechnology: anti-reptin (sc-374135), pontin (sc-393905), Tip60 (sc-166323), RORα1 (sc-26377), RORα3 (sc-38868), and β-actin (sc-8432). The following commercially available antibodies were used: anti-FLAG antibodies (Sigma, F3165), anti-lysine methyl antibodies (Abcam, ab23366), anti-dimethyl histone antibodies (Abcam, ab7766 and ab1220), and anti-RNA Polymerase II antibodies (Berkeley Antibody Company, BioLegend 920401). Anti-RORα2 antibody (target epitope is GKPPYSQKEDKEVQT-C, species: rabbit) was generated by Abmart (China) and immunized eight times with Abmart’s protocol. For Western blot assay, the dilution ratio in 5% skim milk solution was as follows: anti-reptin, pontin, Tip60, and RORα2 for 1:1000; anti-RORα1 and RORα3 for 1:500; anti-β-actin and FLAG for 1:5000.

### 4.2. GST Pulldown Assays

To examine the effect of RORα2 constructs on the binding to pontin and reptin, we firstly prepared GST–RORα2 NTD (Exon 9, 13, and 14) and sp (Exon 13 and 14, specific and different from RORα3) constructs bound to glutathione Sepharose beads. The beads were incubated with the isolated pontin and reptin proteins in a buffer containing 20 mM Tris-HCl (pH 7.5), 150 mM NaCl, 0.2% Nonidet P40, and 10% glycerol. After extensive washing, the bound materials were subjected to Western blot analysis.

### 4.3. Luciferase Reporter Assays

The 293T cells were grown and transiently transfected by using Lipofectamine 2000 reagents (Invitrogen). For luciferase reporter assays, 1 × 10^5^ cells were seeded in DMEM supplemented with 10% FBS for 24 h. Cells were transfected with 200 ng of RORα2E promoter reporter along with 400 ng of other constructs. Using a luciferase assay substrate (Promega: E151A) in the luciferase assay kit (Promega: E1500), the luciferase activity was measured using a luminometer 48 h after transfection and normalized by the expression of beta-galactosidase plasmids. Values are expressed as means ± standard deviations for at least three independent experiments.

### 4.4. Purification and Identification of Binding Proteins for RORα2

RORα2-binding proteins were affinity-purified from extracts of HEK293 cells stably expressing FLAG-pcDNA or FLAG-tagged RORα2. The control and RORα2-binding proteins were immunoprecipitated using anti-FLAG antibody-conjugated agarose beads (80 μL of 50% slurry) from about 90 mg of extracts that were washed with buffer containing 20 mM Tris-HCl (pH 7.9), 15% glycerol, 1 mM EDTA, 1 mM dithiothreitol (DTT), 0.2 mM PMSF, 0.05% Nonidet P40, and 150 mM KCl to remove non-specific contaminants, and the bound materials were eluted by competition with the FLAG peptide (0.1 mg/ml). The bound proteins were resolved by sodium dodecyl sulfate polyacrylamide gel electrophoresis (SDS-PAGE) and prepared for LC–MS/MS analysis.

### 4.5. LC–MS/MS and SEQUEST Analyses

Peptide samples were injected into a column by a Surveyor autosampler (Surveyor, Thermo Finnigan, San Jose, CA) and separated by C18 column. The eluent was directly transferred to the electrospray ionization source of a Thermo Finnigan LCQ DecaXPplus ion trap mass spectrometer. Automated peak recognition, dynamic exclusion, and daughter ion scanning of the two most intense ions were performed and analyzed by the XCALIBUR software. The SEQUEST algorithm was used to interpret MS/MS.

### 4.6. Chromatin Immunoprecipitation (ChIP) 

The ChIP was conducted in LNCaP prostate cancer cells as previously described [50,51] using sheared fragments with an average size of approximately 150 bps. Eluted components were diluted 1:10 with ChIP dilution buffer (20 mM Tris-HCl (pH 8.1), 150 mM NaCl, 2 mM ethylenediaminetetraacetic acid (EDTA), and 1% Triton X-100). Immunoprecipitation was performed using anti-RORα2 (Abmart), dimethyl histone H3K4 (Abcam), dimethyl histone H3K9 (Abcam), polymerase II (Berkeley Antibody Company), and protein A/G beads (SIGMA). For PCR, 1 µL from 50-µL DNA extract and 25–30 cycles of amplification were used. The following primers were used: *CTNND1* promoter (containing RORα2E) sense strand 5’–CCCTGTCTTTCTCTCCTCTCTTTTT–3’, antisense strand 5’–AAGTGATGTCAGCCCCTGTGA–3’; *CTNND1* promoter (control) sense strand 5’–TCAGGGAAAAATAATCCAATCTCAT–3’ and antisense strand 5’–GCTTTCTTCAACATCCCACCAG–3’. 

### 4.7. Cell Proliferation Assay

The number of viable cells in proliferation was measured using a CellTiter 96® AQueous One Solution Cell Proliferation Assay (MTS) (Promega: G3582) according to the manufacturer’s instructions. PC3 cells were seeded in a 96-well culture plate, and, after 48 h, uniform volumes of CellTiter 96® AQueous One Solution Reagent were treated into each well. Cells were incubated for a further hour, followed by reading the amount of soluble formazan produced by cellular reduction of MTS using a plate reader. 

### 4.8. RNA Interference by shRNA of RORα2

The shRNA constructs were made in the context of the mammalian expression vector pcDNA 3.1/myc-HisB that contained a custom-designed multiple cloning site (MCS) cassette. These vectors allow the optimized expression of shRNA constructs. The target sequences of shRNA against RORα2 and Mock shRNA were as follows: shRORα2 (1), 5’–AAGGGAUGAACUUUUUGGGAU–3’; shRORα2. (2) 5’–AAGGGAUGAACUUUUUGGGAU–3’; and shRNA for Mock, 5’–CUGGACUUCCAGAAGAAGAACAUC–3’.

### 4.9. Clonogenic and Tumorigenicity Assay

PC3 cells expressing shMock or shRORα2 were seeded 500 cells/well in a six-well plate for evaluation of colony-forming capability. The medium was changed every two days. After two weeks, colonies were fixed with methanol, followed by staining with 0.25% crystal violet. The plates were photographed and quantified by counting the total number of cells. For experiments examining tumor formation in vivo, a total of 10 million cells with an equal volume of Matrigel (BD Biosciences, Bedford, MA) were injected subcutaneously at the left flank of six-week-old athymic *nu/nu* male mice (Orient, Seoul, Korea). These experiments were carried out with the approval of the Institutional Animal Care and Ethics Committee (SNU-110324-3, 24 March 2011).

### 4.10. Real-Time Q-PCR

The abundance of mRNA was detected by an ABI prism 7300 system with SYBR Green (molecular probes). Primer pairs were designed to amplify 90–150-bp mRNA specific fragments and confirmed as a unique product by melting curve analysis. The PCR conditions were 95 °C (5 min) and 40 cycles of 95 °C (30 s), 56 °C (30 s), and 72 °C (30 s). The quantity of mRNA was calculated using the ΔΔCt method and normalized by using primers to detect HPRT. All reactions were performed as triplicates. Primers (5’–3’) were: hCTNND1, 5’–CCGGGTCTCACCACAAGATC–3’ and 5’–GGGGTCCGTTGAGTTTCAAAT–3’; hHPRT, 5’–TGACACTGGCAAAACAATGCA–3’ and 5’–GGTCCTTTTCACCAGCAAGCT–3’.

### 4.11. Plasmid Construction

RORα2 K81R and K87R plasmids were generated by site-directed mutagenesis using nPfu-Forte DNA polymerase (Enzynomics, Korea). 3X-FLAG-CMV10-RORα2 was used as a template, and oligonucleotides containing each mutation were used as primers. Sense primers used for generation of K81R and K87R were as follows: 5’–GGAGGCAGAATGGCAGGCCACCATATTCAC–3’ and 5’–CCACCATATTCACAAAGGGAAGATAAGGAAGTAC–3’. The amplified fragments were digested with *DpnI* and the ligated plasmids were transformed.

### 4.12. Statistical Analysis

All experiments were performed independently at least three times. Values are expressed as means ± SD. Significance was analyzed using a two-tailed, unpaired *t*-test. A *p*-value of less than 0.05 was considered statistically significant (* *p* < 0.05, ** *p* < 0.01, *** *p* < 0.001).

## Supplementary Materials

Supplementary materials can be found at https://www.mdpi.com/1422-0067/21/5/1622/s1.

## Abbreviations

RORα	Retinoid acid-related orphan receptor α
ONR	Orphan nuclear receptor
NTD	N-terminal domain
PCa	Prostate cancer
DBD	DNA-binding domain
LBD	Ligand-binding domain
