# Supplementary figures and images for: Isoform-Specific Lysine Methylation of RORα2 by SETD7 Is Required for Association of the TIP60 Coactivator Complex in Prostate Cancer Progression
Source: Int J Mol Sci. 2020 Feb 27;21(5):1622. doi: 10.3390/ijms21051622 (PMC7084544; doi:10.3390/ijms21051622)

## Graphical Abstract

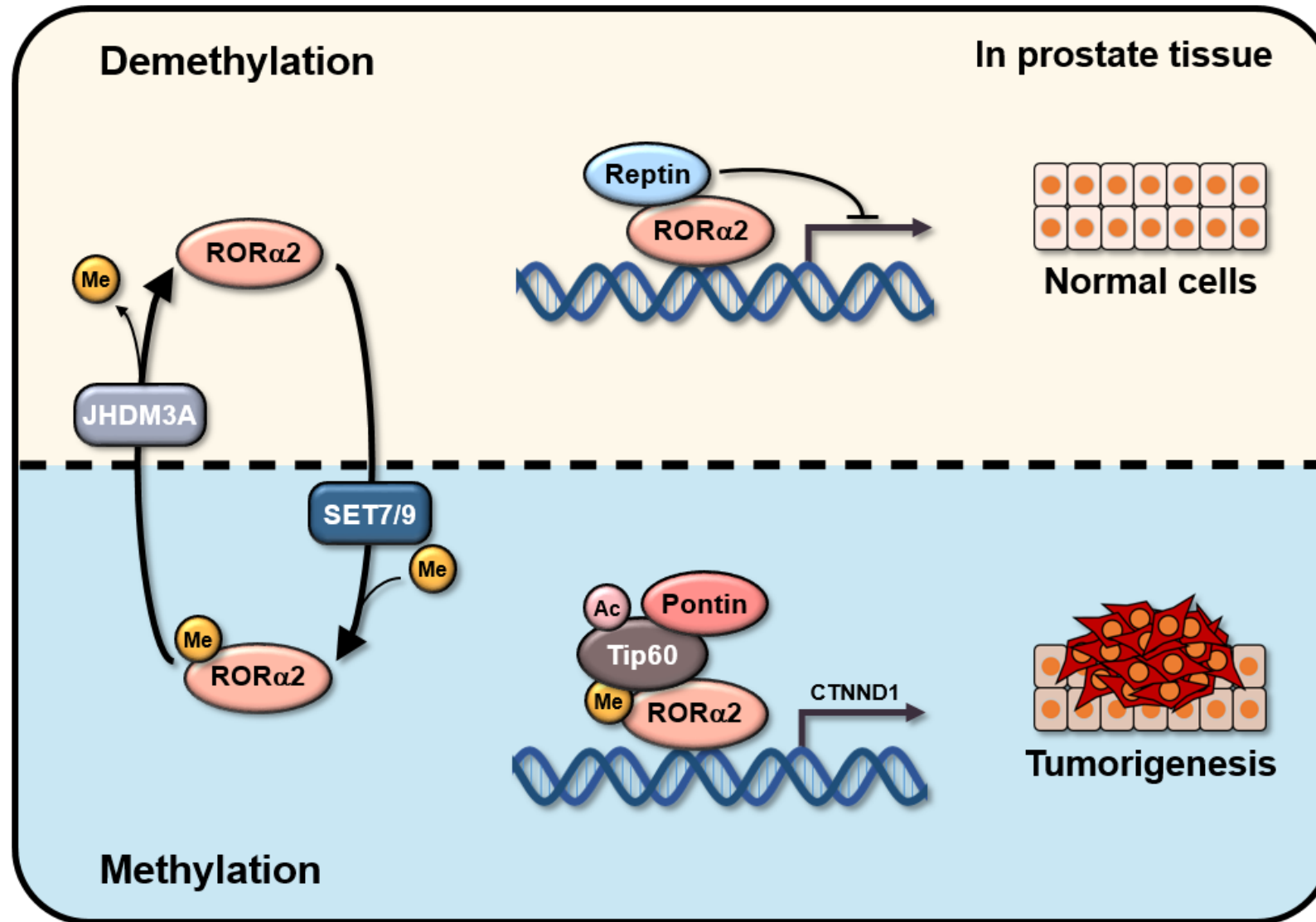

Supplement: Supplementary file 1 [file ijms-21-01622-s001.pdf]
